# Association between dietary inflammatory index and cognitive impairment: A meta-analysis

**DOI:** 10.3389/fnagi.2022.1007629

**Published:** 2023-01-04

**Authors:** Yuxi Jia, Shoumeng Yan, Mengzi Sun, Yixue Yang, Ling Wang, Caihong Wu, Ping Li

**Affiliations:** ^1^Application Demonstration Center of Precision Medicine Molecular Diagnosis, The Second Hospital of Jilin University, Changchun, China; ^2^Department of Orthopedics, The Second Hospital of Jilin University, Changchun, China; ^3^Department of Nursing Humanities, School of Nursing, Jilin University, Changchun, China; ^4^Department of Epidemiology and Biostatistics, School of Public Health, Jilin University, Changchun, China; ^5^Department of Nutrition and Food Hygiene, School of Public Health, Jilin University, Changchun, China; ^6^Department of Developmental Pediatrics, The Second Hospital of Jilin University, Changchun, China

**Keywords:** cognitive impairment, mental disorders, dietary inflammatory index (DII), pro-inflammatory diet, meta-analysis

## Abstract

**Aims:**

Cognitive impairment is an increasingly urgent global public health challenge. Dietary Inflammatory Index (DII) is a literature-derived score that links diet to inflammation. The relationship between DII and cognitive impairment remains controversial. Therefore, our study aimed to analysis the role of DII on the risk of cognitive impairment by meta-analysis.

**Methods:**

PubMed, Cochrane Library, MEDLINE, Web of Science and EMBASE databases were searched up to July 2022. Newcastle–Ottawa scale (NOS) and Joanna Briggs Institute (JBI) Checklist were performed to estimate the quality of studies.

**Results:**

Nine observational studies with 19,379 subjects were included. Our study found that higher DII could elevate the risk of cognitive impairment (OR = 1.46, 95%CI = 1.26, 1.69). Meanwhile, the OR of cognitive impairment was 1.49 (95%CI = 1.21, 1.83) for cross-sectional studies and 1.42 (95%CI = 1.12, 1.79) for cohort studies, respectively.

**Conclusion:**

Our meta-analysis indicated that higher DII (indicating a more pro-inflammatory diet) is related to increased risk of cognitive impairment.

## Introduction

Cognitive impairment is an increasingly urgent global public health challenge, with more than 100 million adults predicted to develop dementia by 2050 (Skoczek-Rubińska et al., 2021a). Cognitive impairment is represented by a series of neurological symptoms, including difficulties with memory, concentration and decision making (Wen et al., 2022). And it has been related to adverse health consequences, including heart disease, poor diabetes control and functional decline in daily activity (Almeida and Flicker, 2001; Mehta et al., 2002; Munshi et al., 2006). Notably, although cognitive impairment does not cause dementia certainly, even mild declines in cognitive function can lead to depression to people (Sartori et al., 2012).

Growing evidence has connected inflammation with cognitive impairment and risk of dementia (Johnson and Godbout, 2007; Godbout and Johnson, 2009). Chronic and excessive inflammatory responses may lead to the progression of cognitive impairment (Sartori et al., 2012). Specially, as a complex set of exposures, diet could affect inflammatory responses cumulatively or interactively, and many foods and nutrients can modulate the inflammatory status both acutely and chronically (de Mello et al., 2011; Khoo et al., 2011; Minihane et al., 2015). For example, inflammation could be increased *via* taking an inflammatory diet which is characterized by high consumption of sweets, fries, red and processed meats, and refined grains (Chen et al., 2019). The Dietary Inflammatory Index (DII), proposed by Shivappa *via* literature review, is a literature-derived score that links diet to inflammation, thus evaluating the potential inflammatory levels of dietary components and providing a comprehensive way to evaluate relationships of inflammatory potential of diet with different health-related outcomes (Shivappa et al., 2014; Charisis et al., 2021).

Previous meta-analysis found that higher DII score was related to symptoms of mental disorder, including depression, anxiety, distress and schizophrenia (Chen et al., 2021). Meanwhile, as a useful tool of dietary inflammatory potential, some studies indicated that DII played a role in the pathophysiology of neurodegenerative diseases (Kheirouri and Alizadeh, 2019). Moreover, although some literatures propose the effect of pro-inflammatory diet on cognitive impairment, the opposite conclusion also exists (Kesse-Guyot et al., 2017; Zabetian-Targhi et al., 2021). The relationship between DII and cognitive impairment remains controversial. Therefore, our study aimed to explore the role of DII on the risk of cognitive impairment by meta-analysis.

## Materials and methods

### Sources and methods of data retrieval

We searched the PubMed, Cochrane Library, MEDLINE, Web of Science and EMBASE databases up to July 2022, and used keywords include dietary inflammatory index, anti-inflammatory diet, pro-inflammatory diet, dietary inflammatory score, DII, cognition, cognitive function, cognitive impairment and cognitive disorder to screen and identify published literatures. The search had no restriction on publication date or language.

### Inclusion criteria

**Figure 1:**
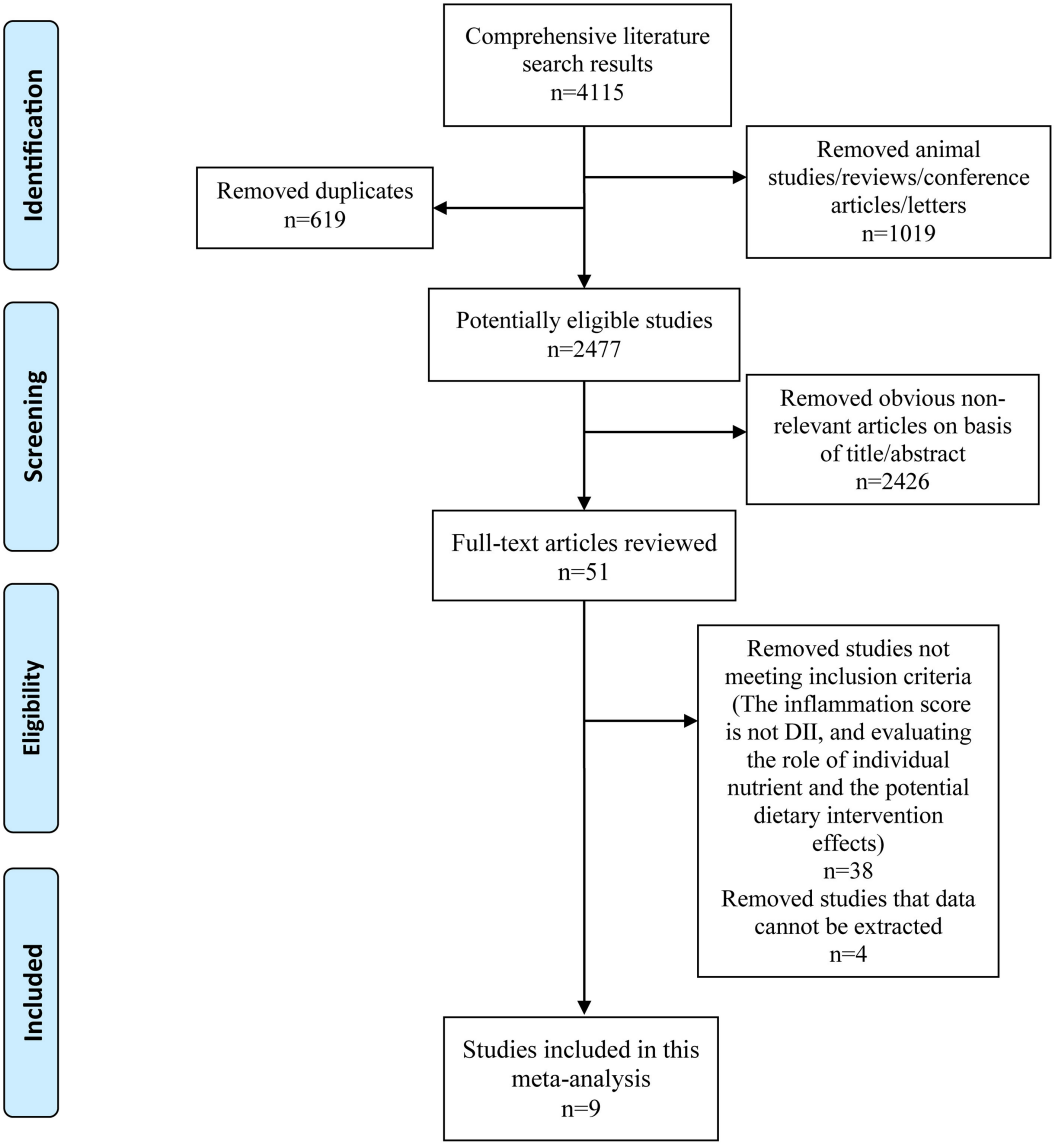
Flow diagram of the literature search and selection.

The inclusion criteria were as follows: (1) observational study including cohort, case–control and cross-sectional design; (2) exposure: inflammatory diet evaluated *via* DII score; (3) effect size for the highest (pro-inflammatory diet) to the lowest (anti-inflammatory diet) DII scores were reported in these studies; (4) occurrence of cognitive impairment as an study outcome; (5) Animal and *in vitro* studies, duplicate and conference literatures, or reviews were excluded. Two authors assessed all studies independently, resolved disagreements *via* discussion, and collected final included studies (Figure 1).

### Data extraction and quality within individual studies

All included studies were examined and following data were collected: first author, study area, publication year, study type, numbers, age, gender and cognitive situation of subjects. Meanwhile, the Newcastle–Ottawa scale (NOS) and the Joanna Briggs Institute (JBI) Checklist were used to evaluate the quality for cohort and cross-sectional study, respectively. Moreover, GRADE system was performed to assess quality of the evidence comments.

### Statistical analysis

All statistical analysis were performed *via* the software Stata (version 12.0, StataCorp LLC, College Station, TX, USA). The *I*^2^ statistic was used to assess the statistical heterogeneity and *P* < 0.05 was defined significant for heterogeneity. In our study, random effects models were used in all analyses based on the level of heterogeneity. Egger's test was performed to evaluate the publication bias, and the trim-and-fill method (sensitivity analysis) was conducted to analysis the influence of bias on the results. Meanwhile, subgroup analyses were performed based on the study area (Europe, Asia and Americas), gender of subjects (women, men and women+men) and study type (cross-sectional study and cohort study).

## Results

A total of 9 studies met inclusion criteria, which contained 19,379 subjects (Hayden et al., 2017; Shin et al., 2018; Charisis et al., 2021; Liu et al., 2021; Skoczek-Rubińska et al., 2021a; Zhang et al., 2021; Song et al., 2022; Sun et al., 2022; Wang et al., 2022). Two studies were performed in Europe, four studies were performed in Asia and three studies were performed in Americas. The study outcomes of all included literatures were varying degrees of cognitive impairment or reduced cognitive function. The NOS are all ≥ 7 for cohort study (*n* = 3) and the JBI are all ≥ 14 for cross-sectional study (*n* = 6) (Table 1). Meanwhile, the GRADE system was conducted to determine the quality of evidence, and the grades of evidence were considered moderate quality (Table 2).

**Table 1:** Characteristics of studies evaluating the association between DII and cognition function.

**References**	**Study area**	**Study type**	**Quality assessment of study (NOS/JBI)**	***n***	**Age (years)**	**Gender**	**Cognition**	**Cognitive function assessment**
Skoczek-Rubińska et al., 2021a	Poland	Cross-sectional study	16 (JBI)	222	61.0 ± 0.4	Women	Cognitive impairment	MMSE
Shin et al., 2018	Korea	Cross-sectional study	14 (JBI)	239	≥65	Women + men	Mild or moderate cognitive impairment	K-MMSE
Hayden et al., 2017	USA	Cohort study	8 (NOS)	7,085	71.0 ± 3.9	Women	MCI or probable dementia	3MS and further clinical evaluation
Sun et al., 2022	USA	Cross-sectional study	19 (JBI)	1,198	≥60	Women + men	Cognitive impairment	CERAD-WL, DSST, AF
Liu et al., 2021	China	Cross-sectional study	18 (JBI)	3,386	≥60	Women + men	MCI	Petersen's criteria
Charisis et al., 2021	Greece	Cohort study	8 (NOS)	1,059	73.1 ± 5.0	Women + men	Dementia	DSM-IV-TR criteria
Song et al., 2022	USA	Cross-sectional study	18 (JBI)	2,901	69.6 ± 6.8	Women/Men	Lower cognitive functioning	CERAD-WL, CERAD-DR, DSST, AF
Zhang et al., 2021	China	Cohort study	7 (NOS)	2,239	58.8 ± 4.7	Women + men	MCI	MMSE, MoCA
Wang et al., 2022	China	Cross-sectional study	15 (JBI)	1,050	65–85	Women + men	MCI	MMSE, MoCA

NOS, Newcastle-Ottawa Scale; JBI, Joanna Briggs Institute (JBI) Checklist; MCI, Mild cognitive impairment; MMSE, Mini-Mental State Examination; K-MMSE, Korean-adjusted version of Mini-Mental State Examination; 3MS, Modified Mini–Mental State Examination; CERAD-WL, Consortium to Establish a Registry for Alzheimer's Disease Word Learning; DSST, Digit Symbol Substitution Test; AF, Animal Fluency test; MoCA, Montreal Cognitive Assessment.

**Table 2:** The summary of findings (SoF) with GRADE system.

**The association between DII and cognitive function**			
Population: Subjects with cognitive impairment vs. normal subjects.			
Settings: Four studies were conducted in Asia, three studies were conducted in Americas, and two studies were conducted in Europe.			
Outcomes	Effect size (95% CI)^a^	No of participants (studies)	Quality of the evidence Comments (GRADE)
Risk of cognitive impairment	1.46 (1.26, 1.69)	19,379 (9 cohort/cross-sectional studies)	⊕⊕⊕ MIDDLE^b^

GRADE working group grades of evidence.

High quality: We are very confident that the true effect lies close to that of the estimate of the effect.

Moderate quality: We are moderately confident in the effect estimate: The true effect is likely to be close to the estimate of the effect, but there is a possibility that it is substantially different.

Low quality: Our confidence in the effect estimate is limited: The true effect may be substantially different from the estimate of the effect.

Very low quality: We have very little confidence in the effect estimate: The true effect is likely to be substantially different from the estimate of effect.

DII, Dietary Inflammatory Index; CI, confidence interval.

^a^Results for Risk of cognitive impairment due to increased DII level.

^b^Upgraded by one level due to all the results of the included studies were almost identical (subjects with cerebrovascular diseases had higher triglyceride glucose index).

**Figure 2:**
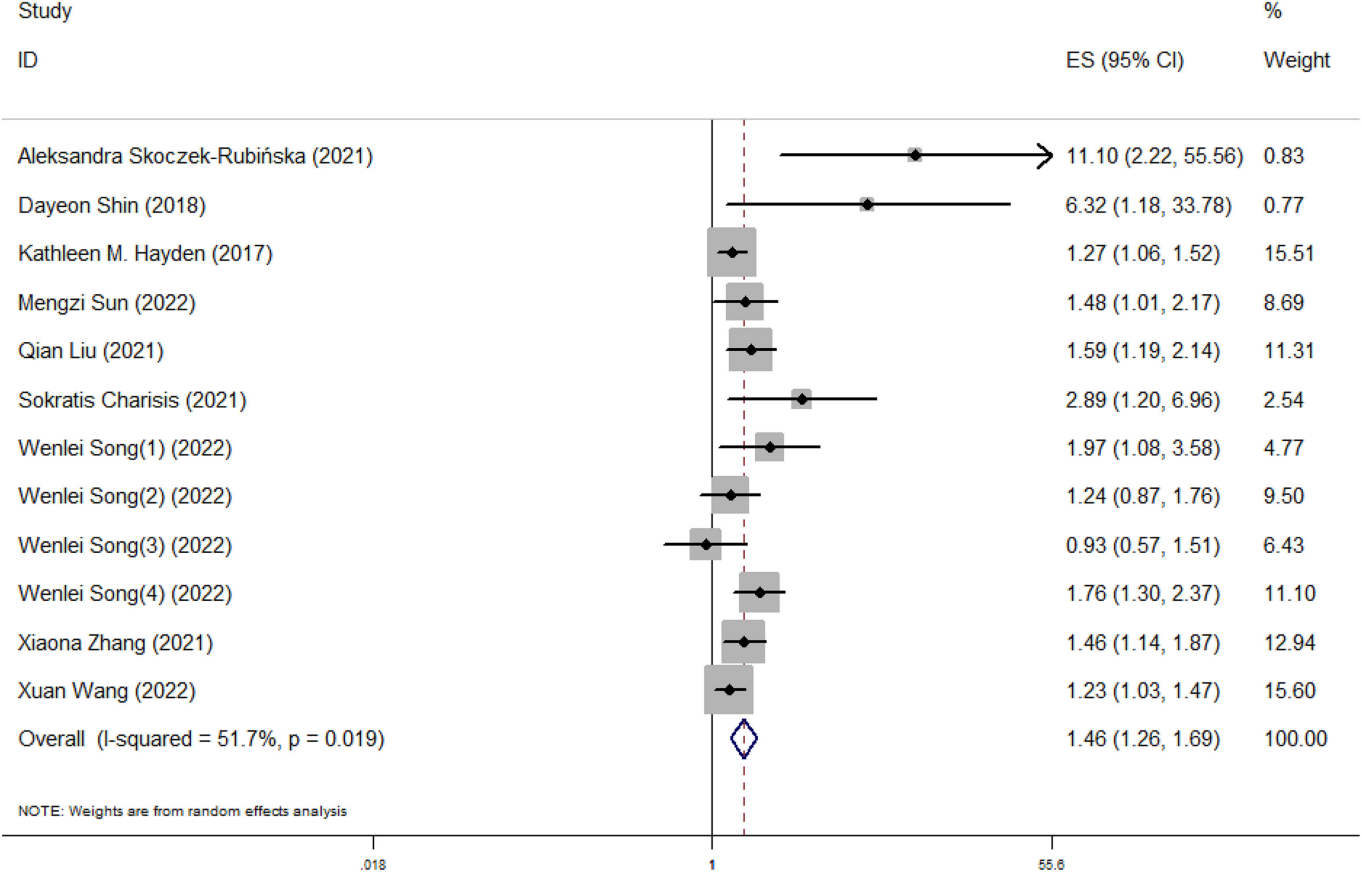
Meta-analysis results of higher DII for the risk of cognitive impairment.

**Figure 3:**
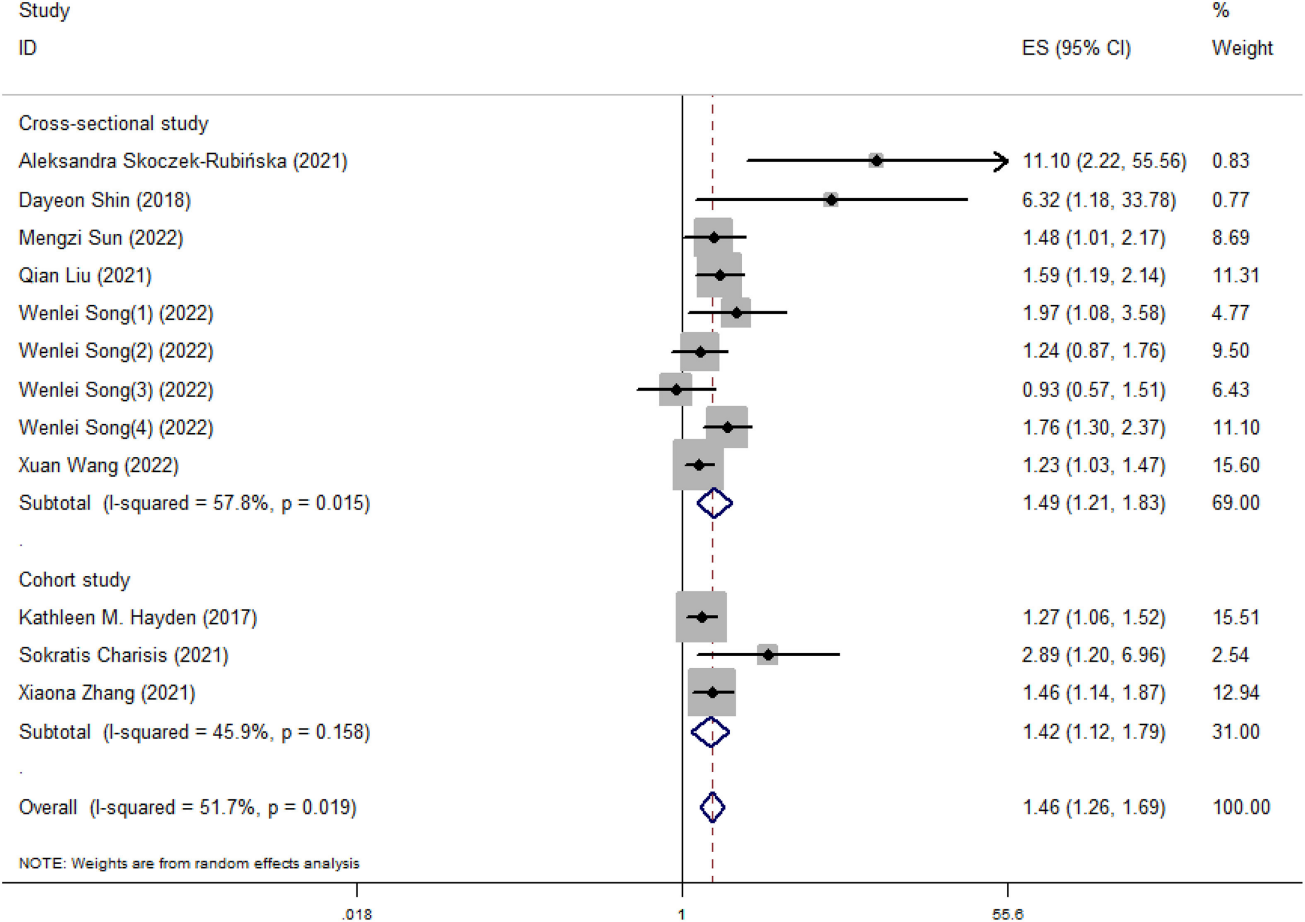
Meta-analysis results of higher DII for the risk of cognitive impairment (subtotals on the basis of study type).

**Figure 4:**
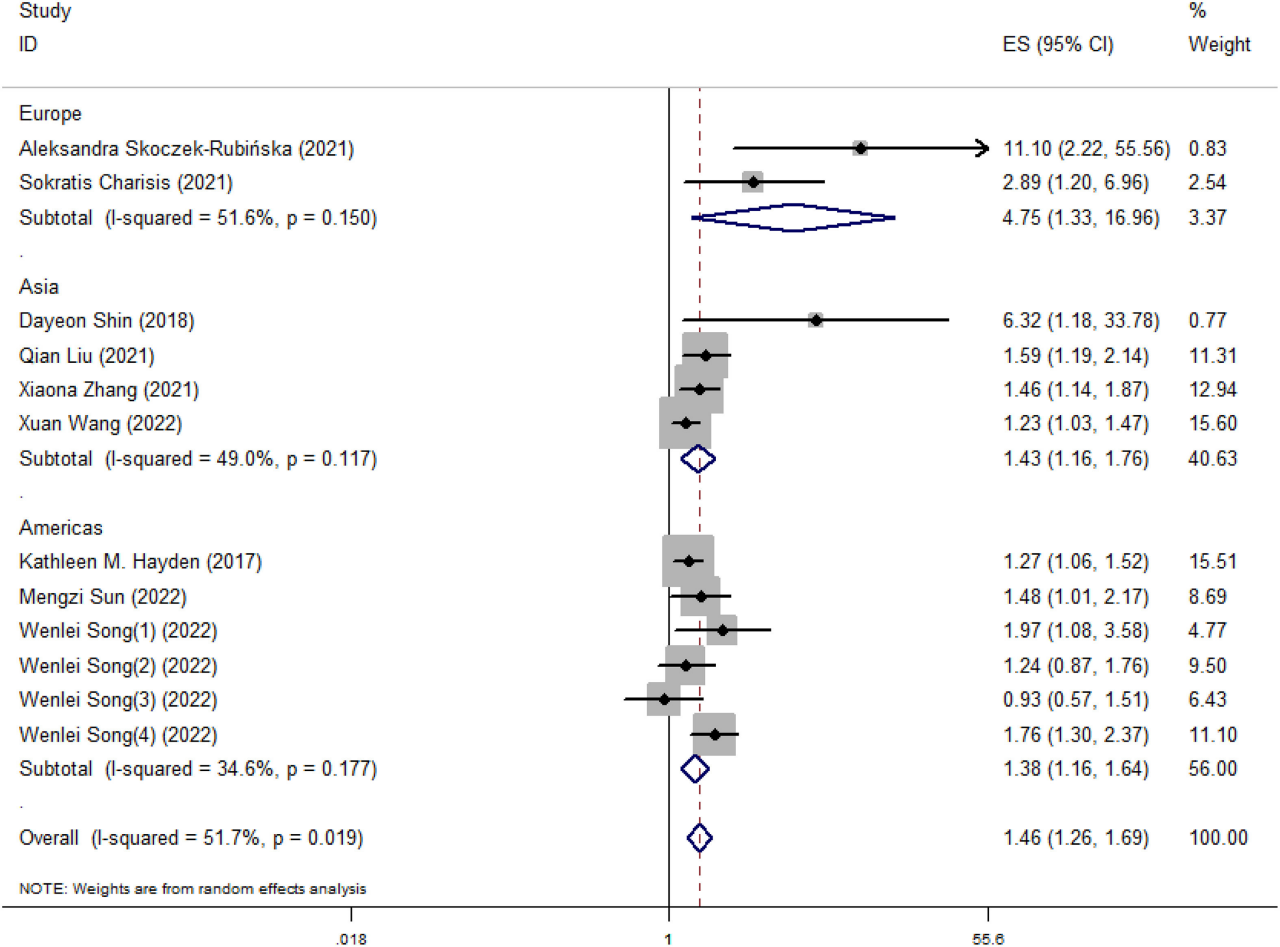
Meta-analysis results of higher DII for the risk of cognitive impairment (subtotals on the basis of study area).

**Figure 5:**
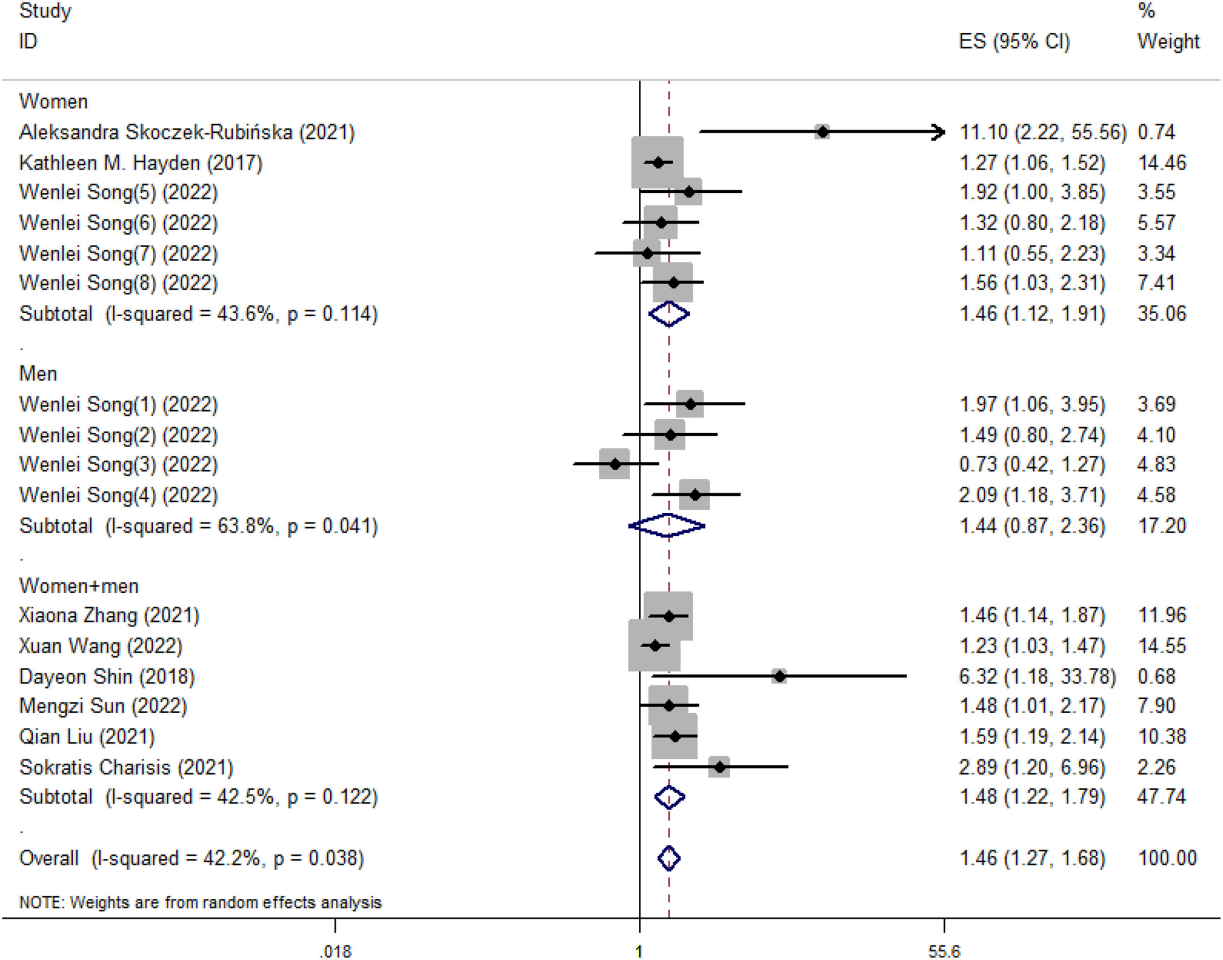
Meta-analysis results of higher DII for the risk of cognitive impairment (subtotals on the basis of the gender of subjects).

Our meta-analysis showed that higher DII scores had a 46% elevated risk of cognitive impairment (OR = 1.46, 95%CI = 1.26, 1.69; Figure 2). In subgroup analysis, a significant positive association between DII and cognitive impairment was found both in cross-sectional and cohort studies (Figure 3). And consistent results were also observed for the subgroup analysis based on study area (Figure 4). In addition, the OR of cognitive impairment for women, men and women+men were 1.46 (95%CI = 1.12, 1.91), 1.44 (95%CI = 0.87, 2.36) and 1.48 (95%CI = 1.22, 1.79), respectively (Figure 5).

**Figure 6:**
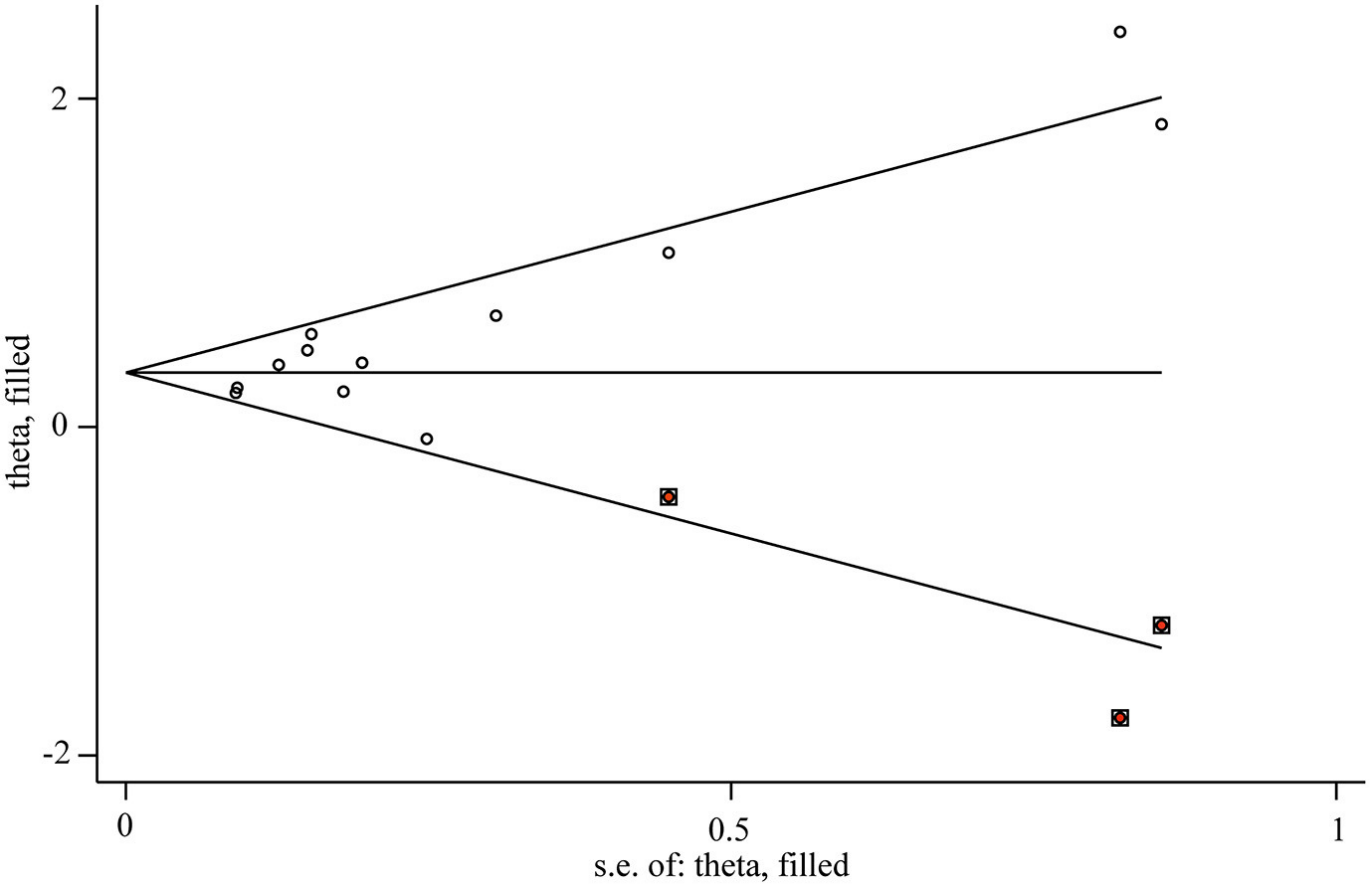
Funnel plot of included studies (after trim and fill).

Publication biases were observed in our meta-analysis (*t* = 3.22, *P* = 0.009). However, there was no significant difference for results after using trim-and-fill method. Therefore, the effect of publication bias was considered slight and the results were stable (Table 3). And the funnel plot after using trim-and-fill method was performed in Figure 6.

**Table 3:** Publication bias (Egger test) and sensitivity analysis (method of trim and fill) for included studies.

**Original variation**
OR (95%CI)	Egger test (*t, P*)
1.46 (1.26, 1.69)	3.22, 0.009
**Variation after trim and fill**
OR (95%CI)	Number of trim and fill
1.39 (1.17, 1.66)	3

## Discussion

Continuously elevated levels of inflammation are related to neurodegeneration, atherosclerotic processes, and chronic diseases (Paul et al., 2004; Raz and Rodrigue, 2006; Russo et al., 2011). Meanwhile, inflammation has been closely linked to diet (Giugliano et al., 2006). Higher DII scores, also known as larger inflammatory potential of the diet, have been related to increased levels of inflammatory biomarker including IL-6, CRP, and TNF-α, thus linking to cognitive impairment (Hayden et al., 2017; Shin et al., 2018). Our meta-analysis found similar results.

Diet is now recognized as an important factor in modifying inflammation (Li et al., 2021). Evidence suggests that a pro-inflammatory diet could elevate levels of inflammatory cytokines through oxidative stress and immune mechanisms (Li et al., 2021). After ingesting a pro-inflammatory diet, free radicals were produced *via* macrophages and were released into tissues, thereby promoting cell oxidative stress. Meanwhile, a pro-inflammatory diet could also disturb the integrity of the intestinal immune cell barrier and cause intestinal cytotoxic effects, further producing effects on immune function (Li et al., 2021). Chronically inflamed gut leads to systemic immunologic activation and further promotes neuroinflammation, and triggering cognitive decline and dementia (Daulatzai, 2014). Correspondingly, a variety of inflammation-related proteins including LPS, complement factors, acute-phase proteins, and pro-inflammatory cytokines increase in the brains (Zhang et al., 2009). Subsequently, long-term inflammation could damage the blood-brain barrier, and some inflammatory cytokines (IL-1β, IL-6 and TNF-α) can cross the blood-brain barrier elevating neuroinflammation, thus leading to cognitive impairment (Engelhart et al., 2004; Heneka et al., 2015; d'Avila et al., 2018; Godos et al., 2020). For example, high levels of IL-1β are detected in microglial cells surrounding amyloid β (Aβ) plaques in Alzheimer's disease (AD) patient brains. Meanwhile, *in vitro*, IL-1β can favor Aβ deposition by modulating APP expression and proteolysis. And pathological accumulation of Aβ is a key factor that drives neuroinflammatory responses in AD (Heneka et al., 2015). In addition, overexpression of TNF-a has been proved to trigger chronic central nervous system inflammation and white matter degeneration (Probert et al., 1997). Meanwhile, as a major regulator of inflammatory response in the central nervous system, microglia is critical for maintaining brain homeostasis, and its activation is both characterized and modulated by above cytokines (Gomez-Nicola and Perry, 2015; Heneka et al., 2015). Evidence indicated that aging microglia could overreact when acute inflammation occurs, and the changes may further affect cognitive function (d'Avila et al., 2018). Notably, clinical studies have documented increased incidence of memory loss in inflammatory bowel disease (IBD) patients, attention deficits and declining executive functions. The potential mechanism may also be related to hippocampal neurogenesis and local innate immune response (Gampierakis et al., 2021). In a word, there is an intriguing interaction between the gut, brain and the immune systems, while any dysregulation in this communication is considered to affect the balance between central nervous system (CNS) homeostasis and neuropathology (Bonaz and Bernstein, 2013).

Symptoms of cognitive impairment range from mild to severe, and the prevalence varies with age, gender and geographical location (Wen et al., 2022). Our meta-analysis performed subgroup analysis based on study area and gender of subjects. Different regions have different dietary habits. For example, in some Asian countries, the diet is carbohydrate-based and mainly consists of rice (Park et al., 2010). And excessive intake of refined carbohydrates has been related to higher risks for cognitive impairment (Alley et al., 2008; Marioni et al., 2010; Trollor et al., 2012). In addition, the results of subgroup analysis based on gender of subjects indicated that the significant relationship between DII and cognitive impairment was only found in women, which was consistent with Shin's research (Shin et al., 2018). Meanwhile, previous studies have found that unhealthy dietary habits were found among postmenopausal women (Ryu et al., 2019; Skoczek-Rubińska et al., 2021b). One possible reason is that appetite-control mechanisms in hypothalamus become imbalanced in the postmenopausal state, leading to increased high-fat and high-carbohydrate foods intake, thus causing inflammation (Christensen and Pike, 2015; Stachowiak et al., 2015; Kozakowski et al., 2017).

In our meta-analysis, we noted that some included studies assessed the relationship between DII and mild cognitive impairment (MCI). MCI is a transitional state between healthy aging and dementia, characterized by cognitive decline but relatively complete activities of daily living (Anderson, 2019). The prevalence of MCI in elderly is 6.7–25.2% (Jongsiriyanyong and Limpawattana, 2018). And people with MCI have an higher risk of dementia, with an annual rate of growth in 10–15% (Eshkoor et al., 2015). Notably, in fact, brain lesions occur long before cognitive symptoms appear, and could be irreversibly altered by the time of diagnosis (Sperling et al., 2013). Therefore, many researchers have turned their attention to people in the preclinical stages of the disease. And MCI could be viewed as a “window” in which it could be access to intervene and postpone development to dementia (Anderson, 2019). More studies on DII and MCI is needed in the future to evaluate their relationship.

Some limitations are existed in our study. All included literatures are all observational study, it is difficult to clear the cause–effect relationship of DII on cognitive impairment. Meanwhile, although all results of original studies were almost identical, the dose-response association between DII and cognitive impairment could not be evaluated due to lack of corresponding data. Therefore, the true effect is likely to be close to the estimated effect, but the possibility of a difference exists. In addition, due to limited original studies, we were unable to perform subgroup analysis on the age and level of cognitive impairment of subjects. More data are needed to assess the role of these factors.

## Conclusion

Our meta-analysis indicated that higher DII (representing a more pro-inflammatory diet) could increase the risk of cognitive impairment. More data from clinical trials are needed to verify the association.

## Data availability statement

The original contributions presented in the study are included in the article/supplementary material, further inquiries can be directed to the corresponding author.

## Author contributions

PL and YJ designed the study. PL, YJ, SY, MS, and CW performed the study. YJ and YY analyzed the data and wrote the manuscript. YJ and LW participated in revising the manuscript. All authors agreed with the final version of the manuscript.

## References

[B1] Alley D. E. Crimmins E. M. Karlamangla A. Hu P. Seeman T. E. (2008). Inflammation and rate of cognitive change in high-functioning older adults. J. Gerontol. A. Biol. Sci. Med. Sci. 63, 50–55. 10.1093/gerona/63.1.50 18245760 PMC2952346

[B2] Almeida O. P. Flicker L. (2001). The mind of a failing heart: a systematic review of the association between congestive heart failure and cognitive functioning. Intern. Med. J. 31, 290–295. 10.1046/j.1445-5994.2001.00067.x 11512600

[B3] Anderson N. D. (2019). State of the science on mild cognitive impairment (MCI). CNS Spectr. 24, 78–87. 10.1017/S1092852918001347 30651152

[B4] Bonaz B. L. Bernstein C. N. (2013). Brain-gut interactions in inflammatory bowel disease. Gastroenterology 144, 36–49. 10.1053/j.gastro.2012.10.003 23063970

[B5] Charisis S. Ntanasi E. Yannakoulia M. Anastasiou C. A. Kosmidis M. H. Dardiotis E. . (2021). Diet inflammatory index and dementia incidence. A population-based study. Neurology 97, e2381–e23891. 10.1212/WNL.0000000000012973 34759053 PMC8673721

[B6] Chen G. Q. Peng C. L. Lian Y. Wang B. W. Chen P. Y. Wang G. P. (2021). Association between dietary inflammatory index and mental health: a systematic review and dose-response meta-analysis. Front Nutr. 8, 662357. 10.3389/fnut.2021.662357 34026809 PMC8133218

[B7] Chen X. Maguire B. Brodaty H. O'Leary F. (2019). Dietary patterns and cognitive health in older adults: a systematic review. J. Alzheimer's Dis. 67, 583–619. 10.3233/JAD-180468 30689586

[B8] Christensen A. Pike C. J. (2015). Menopause, obesity and inflammation: interactive risk factors for Alzheimer's disease. Front. Aging Neurosci. 7, 130. 10.3389/fnagi.2015.00130 26217222 PMC4493396

[B9] Daulatzai M. A. (2014). Chronic functional bowel syndrome enhances gut-brain axis dysfunction, neuroinflammation, cognitive impairment, and vulnerability to dementia. Neurochem. Res. 39, 624–644. 10.1007/s11064-014-1266-6 24590859

[B10] d'Avila J. C. Siqueira L. D. Mazeraud A. Azevedo E. P. Foguel D. Castro-Faria-Neto H. C. . (2018). Age-related cognitive impairment is associated with long-term neuroinflammation and oxidative stress in a mouse model of episodic systemic inflammation. J. Neuroinflammation. 15, 28. 10.1186/s12974-018-1059-y 29382344 PMC5791311

[B11] de Mello V. D. Schwab U. Kolehmainen M. Koenig W. Siloaho M. Poutanen K. . (2011). A diet high in fatty fish, bilberries and wholegrain products improves markers of endothelial function and inflammation in individuals with impaired glucose metabolism in a randomised controlled trial: the Sysdimet study. Diabetologia. 54, 2755–2767. 10.1007/s00125-011-2285-3 21870174

[B12] Engelhart M. J. Geerlings M. I. Meijer J. Kiliaan A. Ruitenberg A. van Swieten J. C. . (2004). Inflammatory proteins in plasma and the risk of dementia: the rotterdam study. Arch. Neurol. 61, 668–672. 10.1001/archneur.61.5.668 15148142

[B13] Eshkoor S. A. Hamid T. A. Mun C. Y. Ng C. K. (2015). Mild cognitive impairment and its management in older people. Clin. Interv. Aging. 10, 687–693. 10.2147/CIA.S73922 25914527 PMC4401355

[B14] Gampierakis I. A. Koutmani Y. Semitekolou M. Morianos I. Polissidis A. Katsouda A. . (2021). Hippocampal neural stem cells and microglia response to experimental inflammatory bowel disease (IBD). Mol. Psychiatry. 26, 1248–1263. 10.1038/s41380-020-0651-6 31969694

[B15] Giugliano D. Ceriello A. Esposito K. (2006). The effects of diet on inflammation: emphasis on the metabolic syndrome. J. Am. Coll. Cardiol. 48, 677–685. 10.1016/j.jacc.2006.03.052 16904534

[B16] Godbout J. P. Johnson R. W. (2009). Age and neuroinflammation: a lifetime of psychoneuroimmune consequences. Immunol Allergy Clin North. 29, 321–337. 10.1016/j.iac.2009.02.007 19389585

[B17] Godos J. Currenti W. Angelino D. Mena P. Castellano S. Caraci F. . (2020). Diet and mental health: review of the recent updates on molecular mechanisms. Antioxidants 9, 346. 10.3390/antiox9040346 32340112 PMC7222344

[B18] Gomez-Nicola D. Perry V. H. (2015). Microglial dynamics and role in the healthy and diseased brain: a paradigm of functional plasticity. Neuroscientist 21, 169–84. 10.1177/1073858414530512 24722525 PMC4412879

[B19] Hayden K. M. Beavers D. P. Steck S. E. Hebert J. R. Tabung F. K. Shivappa N. . (2017). The association between an inflammatory diet and global cognitive function and incident dementia in older women: the women's health initiative memory study. Alzheimers Dement. 13, 1187–1196. 10.1016/j.jalz.2017.04.004 28531379 PMC5909961

[B20] Heneka M. T. Carson M. J. El Khoury J. Landreth G. E. Brosseron F. Feinstein D. L. . (2015). Neuroinflammation in Alzheimer's disease. Lancet Neurol. 14, 388–405. 10.1016/S1474-4422(15)70016-5 25792098 PMC5909703

[B21] Johnson R. W. Godbout J. P. (2007). “Aging, neuroinflammation, and behavior,” in Psychoneuroimmunology, 4th Edn. p. 379–391. 10.1016/B978-012088576-3/50022-8

[B22] Jongsiriyanyong S. Limpawattana P. (2018). Mild cognitive impairment in clinical practice: a review article. Am. J. Alzheimers Dis. Other Demen. 33, 500–507. 10.1177/1533317518791401 30068225 PMC10852498

[B23] Kesse-Guyot E. Assmann K. E. Andreeva V. A. Touvier M. Neufcourt L. Shivappa N. . (2017). Long-term association between the dietary inflammatory index and cognitive functioning: findings from the SU.VI.MAX study. Eur. J. Nutr. 56, 1647–1655. 10.1007/s00394-016-1211-3 27055851

[B24] Kheirouri S. Alizadeh M. (2019). Dietary inflammatory potential and the risk of neurodegenerative diseases in adults. Epidemiol. Rev. 41, 109–120. 10.1093/epirev/mxz005 31565731

[B25] Khoo J. Piantadosi C. Duncan R. Worthley S. G. Jenkins A. Noakes M. . (2011). Comparing effects of a low-energy diet and a high-protein low-fat diet on sexual and endothelial function, urinary tract symptoms, and inflammation in obese diabetic men. J. Sex. Med. 8, 2868–2875. 10.1111/j.1743-6109.2011.02417.x 21819545

[B26] Kozakowski J. Gietka-Czernel M. Leszczyńska D. Majos A. (2017). Obesity in menopause - our negligence or an unfortunate inevitability? Prz. Menopauzalny 16, 61–65. 10.5114/pm.2017.68594 28721132 PMC5509974

[B27] Li R. Zhan W. Huang X. Liu Z. Lv S. Wang J. . (2021). Association of dietary inflammatory index (DII) and depressive disorders. J. Inflamm. Res. 14, 6959–6973. 10.2147/JIR.S344002 34949933 PMC8691198

[B28] Liu Q. Zhou D. Duan H. Zhu Y. Du Y. Sun C. . (2021). Association of dietary in?ammatory index and leukocyte telomere length with mild cognitive impairment in Chinese older adults. Nutr. Neurosci. 26, 1–10. 10.1080/1028415X.2021.2017660 34957928

[B29] Marioni R. E. Strachan M. W. Reynolds R. M. Lowe G. D. Mitchell R. J. Fowkes F. G. . (2010). Association between raised inflammatory markers and cognitive decline in elderly people with type 2 diabetes: the Edinburgh Type 2 Diabetes Study. Diabetes 59, 710–713. 10.2337/db09-1163 19959761 PMC2828661

[B30] Mehta K. M. Yaffe K. Covinsky K. E. (2002). Cognitive impairment, depressive symptoms, and functional decline in older people. J. Am. Geriatr. Soc. 50, 1045–1050. 10.1046/j.1532-5415.2002.50259.x 12110064 PMC2939718

[B31] Minihane A. M. Vinoy S. Russell W. R. Baka A. Roche H. M. Tuohy K. M. . (2015). Low-grade inflammation, diet composition and health: current research evidence and its translation. Br. J. Nutr. 114, 999–1012. 10.1017/S0007114515002093 26228057 PMC4579563

[B32] Munshi M. Grande L. Hayes M. Ayres D. Suhl E. Capelson R. . (2006). Cognitive dysfunction is associated with poor diabetes control in older adults. Diabetes Care. 29, 1794–1799. 10.2337/dc06-0506 16873782 PMC1615865

[B33] Park S. H. Lee K. S. Park H. Y. (2010). Dietary carbohydrate intake is associated with cardiovascular disease risk in Korean: analysis of the third Korea National Health and Nutrition Examination Survey (KNHANES III). Int. J. Cardiol. 139, 234–240. 10.1016/j.ijcard.2008.10.011 19013653

[B34] Paul A. Ko K. W. Li L. Yechoor V. McCrory M. A. Szalai A. J. . (2004). C-reactive protein accelerates the progression of atherosclerosis in apolipoprotein E-deficient mice. Circulation 109, 647–655. 10.1161/01.CIR.0000114526.50618.24 14744975

[B35] Probert L. Akassoglou K. Kassiotis G. Pasparakis M. Alexopoulou L. Kollias G. (1997). TNF-alpha transgenic and knockout models of CNS inflammation and degeneration. J. Neuroimmunol. 72, 137–141. 10.1016/S0165-5728(96)00184-1 9042105

[B36] Raz N. Rodrigue K. M. (2006). Differential aging of the brain: patterns, cognitive correlates and modifiers. Neurosci. Biobehav. Rev. 30, 730–748. 10.1016/j.neubiorev.2006.07.001 16919333 PMC6601348

[B37] Russo I. Barlati S. Bosetti F. (2011). Effects of neuroinflammation on the regenerative capacity of brain stem cells. J. Neurochem. 116, 947–956. 10.1111/j.1471-4159.2010.07168.x 21198642 PMC3382108

[B38] Ryu I. Kwon M. Sohn C. Shivappa N. Hébert J. R. Na W. . (2019). The association between dietary inflammatory index (DII) and cancer risk in Korea: a prospective cohort study within the KoGES-HEXA study. Nutrients. 11, 2560. 10.3390/nu11112560 31652856 PMC6893737

[B39] Sartori A. C. Vance D. E. Slater L. Z. Crowe M. (2012). The impact of inflammation on cognitive function in older adults: implications for healthcare practice and research. J. Neurosci. Nurs. 44, 206–217. 10.1097/JNN.0b013e3182527690 22743812 PMC3390758

[B40] Shin D. Kwon S. C. Kim M. H. Lee K. W. Choi S. Y. Shivappa N. . (2018). Inflammatory potential of diet is associated with cognitive function in an older adult Korean population. Nutrition 55–56:56–62. 10.1016/j.nut.2018.02.026 29960158 PMC8684699

[B41] Shivappa N. Steck S. E. Hurley T. G. Hussey J. R. Hébert J. R. (2014). Designing and developing a literature-derived, population-based dietary inflammatory index. Public Health Nutr. 17, 1689–1696. 10.1017/S1368980013002115 23941862 PMC3925198

[B42] Skoczek-Rubińska A. Muzsik-Kazimierska A. Chmurzynska A. Jamka M. Walkowiak J. Bajerska J. (2021a). Inflammatory potential of diet is associated with biomarkers levels of inflammation and cognitive function among postmenopausal women. Nutrients 13, 2323. 10.3390/nu13072323 34371834 PMC8308633

[B43] Skoczek-Rubińska A. Muzsik-Kazimierska A. Chmurzynska A. Walkowiak P. J. Bajerska J. (2021b). Snacking may improve dietary fiber density and is associated with a lower body mass index in postmenopausal women. Nutrition 83, 111063. 10.1016/j.nut.2020.111063 33352354

[B44] Song W. Feng Y. Gong Z. Tian C. (2022). The association between dietary inflammatory index and cognitive performance in older adults aged 60 years and older. Front Nutr. 9, 748000. 10.3389/fnut.2022.748000 35495906 PMC9039302

[B45] Sperling R. A. Karlawish J. Johnson K. A. (2013). Preclinical Alzheimer disease-the challenges ahead. Nat Rev. Neurol. 9, 54–58. 10.1038/nrneurol.2012.241 23183885 PMC3643203

[B46] Stachowiak G. Pertyński T. Pertyńska-Marczewska M. (2015). Metabolic disorders in menopause. Prz. Menopauzalny 14, 59–64. 10.5114/pm.2015.50000 26327890 PMC4440199

[B47] Sun M. Wang L. Guo Y. Yan S. Li J. Wang X. . (2022). The association among inflammatory diet, glycohemoglobin, and cognitive function impairment in the elderly: based on the NHANES 2011-2014. J. Alzheimer's Dis. 87, 1713–1723. 10.3233/JAD-215688 35491786

[B48] Trollor J. N. Smith E. Agars E. Kuan S. A. Baune B. T. Campbell L. . (2012). The association between systemic inflammation and cognitive performance in the elderly: the sydney memory and ageing study. Age. 34, 1295–1308. 10.1007/s11357-011-9301-x 21853262 PMC3448981

[B49] Wang X. Li T. Li H. Li D. Wang X. Zhao A. . (2022). Association of dietary inflammatory potential with blood inflammation: the prospective markers on mild cognitive impairment. Nutrients 14, 2417. 10.3390/nu14122417 35745147 PMC9229190

[B50] Wen S. Elias P. M. Wakefield J. S. Mauro T. M. Man M. Q. (2022). The link between cutaneous inflammation and cognitive impairment. J. Eur. Acad. Dermatol. Venereol. 36, 1705–1712. 10.1111/jdv.18360 35748522 PMC9481668

[B51] Zabetian-Targhi F. Srikanth V. K. Smith K. J. Oddy Ph D. W. Beare R. Moran C. . (2021). Associations between the dietary inflammatory index, brain volume, small vessel disease, and global cognitive function. J. Acad. Nutr. Diet. 121, 915–924.e3. 10.1016/j.jand.2020.11.004 33339764

[B52] Zhang R. Miller R. G. Gascon R. Champion S. Katz J. Lancero M. . (2009). Circulating endotoxin and systemic immune activation in sporadic amyotrophic lateral sclerosis (sALS). J. Neuroimmunol. 206, 121–124. 10.1016/j.jneuroim.2008.09.017 19013651 PMC2995297

[B53] Zhang X. Wang Y. Liu W. Wang T. Wang L. Hao L. . (2021). Diet quality, gut microbiota, and microRNAs associated with mild cognitive impairment in middle-aged and elderly Chinese population. Am. J. Clin. Nutr. 114, 429–440. 10.1093/ajcn/nqab078 33871591

